# Editorial: Interactive dynamics of geminiviruses with host plants

**DOI:** 10.3389/fpls.2025.1616024

**Published:** 2025-05-29

**Authors:** Muhammad Naeem Sattar

**Affiliations:** Central Laboratories, King Faisal University, Al-Hafuf, Saudi Arabia

**Keywords:** geminiviruses, host-pathogen interactions, plant immunity, viral pathogenesis, Bemisia tabaci, disease management

Geminiviruses, members of the *Geminiviridae* family characterized by their unique twinned icosahedral particles, pose a significant threat to global agriculture. Geminiviruses infest numerous crops such as tomatoes, cassava, cotton, and beans that result in huge losses to agriculture as well as in matters related to food security, more so in tropical and subtropical areas. The complex interplay between geminiviruses and their hosts is an intriguing but complex research area, ranging from molecular to cellular to ecological intersections. This Research Topic, “Interactive Dynamics of Geminiviruses with Host Plants”, compiled by Drs. Muhammad Naeem Sattar, Zahir Ali, and Jesus Mendez-Lozano, included seven research articles and one review article. The articles in this Research Topic address different facets in relation to geminivirus interactions with their hosts. These discussed the complex relationship between geminiviruses with their plant hosts ranging from their efficient diagnosis, functional characterization to controlling them efficiently.

Initial detection of geminiviruses often relies on observing characteristic symptoms in infected plants, such as leaf curling, mosaic patterns, and stunting. However, molecular approaches play an important role in definite identification as well as complete characterization. Atik and Paylan highlights the importance of seed testing and updating quarantine lists to control various viral pathogens. They identified the presence of tobacco mosaic virus (TMV) in 12% of the seeds, tomato mosaic virus (ToMV) in 23%, and cucumber mosaic virus (CMV) in 8% of the seeds, including instances of mixed infections. The findings underscore the necessity for developing robust seed health testing and the use of new generation diagnostics tools to effectively manage and control viral diseases. This was further highlighted by the findings from Sattar et al. The research discovered several distinct begomoviruses in the Al-Ahsa region of Saudi Arabia using high-throughput sequencing technique. They further highlighted the importance of continuous disease monitoring and understanding the evolutionary dynamics of begomoviruses to provide crucial information for effective disease management. The key findings of Yoboué et al. revealed specific alternative weed hosts that show diverse interactions with different cassava mosaic begomoviruses (CMBs) species, suggesting their key role in the emergence of new viruses in cassava. Several studies focus on the role of *Bemisia tabaci*, the whitefly vector, in shaping virus evolution and spread. The diversity of *B. tabaci* biotypes and their varying transmission efficiencies underscore the importance of vector biology in geminivirus epidemiology. Using RNA interference (RNAi) technique, Thesnim et al. identified TLR3 and TOB1 as potentially new genetic targets that could help control *B. tabaci* populations and reduce the transmission of chili leaf curl virus (ChiLCV). Other contributions examine the genetic and epigenetic changes in host plants during infection, providing insights into how viruses manipulate host gene expression and chromatin structure to their advantage.

Geminiviruses are masters of manipulation, exploiting host cellular machinery to replicate and spread. The small, single-stranded DNA genomes of geminiviruses and their associated DNA-satellites encode a limited number of proteins, yet these proteins exhibit remarkable functional versatility. Viral proteins such as TrAP interact with host factors to hijack the plant’s transcriptional and replication machinery. Meanwhile, proteins like C4 and V2 play critical roles in suppressing host immune responses, including RNA silencing and hormone-mediated defenses. Understanding these interactions at the molecular level is crucial for developing targeted strategies to disrupt viral infection cycles. Gupta et al. discovered a previously unknown βV1 protein, originating from the radish leaf curl betasatellite (RaLCB). This protein functions as both an elicitor, inducing a hypersensitive response (HR) that causes cell death in plants, and a virulence factor that may promote viral infection by interacting with the replication enhancer protein (AC3) of the helper virus within the nucleus. They highlighted that betasatellites associated with begomoviruses can encode proteins that actively contribute to disease development not only by enhancing viral replication but also by directly triggering plant defense responses that ultimately cause damage. This highlights the complex ways in which these viral components manipulate host plants to their advantage. Host plants, on the other hand, are not passive victims. They have evolved sophisticated defense mechanisms to detect and counteract viral infections. Siskos et al. identified the host plant’s DNA Primase Large subunit (PRiL) as a crucial factor required for the replication of geminiviruses. Resistance to tomato leaf curl New Delhi virus (ToLCNDV) in melon was found to be consistently associated with a specific mutation in the PRiL gene. Furthermore, silencing the PRiL gene in *Nicotiana benthamiana* significantly reduced the levels of three different geminiviruses, confirming PRiL’s essential role in their multiplication. The study proposes a model where PRiL, like its function in host DNA replication, helps generate the initial RNA primer needed for the conversion of the viral single-stranded DNA into the double-stranded form, a critical first step in geminivirus replication. Understanding such interactions may unveil new paths for resistance development to these common and destructive plant viruses by targeting this particular host protein.

Integrated strategies are crucial for combating geminiviruses, as highlighted in this Research Topic. Traditional methods, such as breeding for resistance, have shown promise but are often undermined by the rapid evolution of viral strains. The review article by Nalla et al. highlighted the need for multidisciplinary strategies combining traditional breeding with advanced genomic technologies to develop and deploy resistant chili cultivars across diverse agricultural settings, ultimately aiming to improve the sustainability and profitability of chili production, particularly against ChiLCV. Al-Roshdi et al. aimed to increase TYLCV tolerance in tomatoes by targeting the AC1/Rep gene. This suggests that RNA interference (RNAi)-based approaches could be a promising strategy for developing virus-resistant tomato varieties, especially in regions like Oman where natural resistance is lacking.

As we continue to unravel the interactive dynamics of geminiviruses with their host plants, interdisciplinary collaboration is essential. Combining insights from molecular biology, genetics, ecology, and agronomy will enable us to develop sustainable strategies for managing geminivirus diseases. This Research Topic aims to inspire further exploration of this critical area, fostering innovation and collaboration among researchers, breeders, and policymakers.

We sincerely thank the writers, peer reviewers, and editorial team for their valuable contributions to this Research Topic. Their hard work has deepened our knowledge of geminivirus-host relationships and paved the way for further studies. We hope this collection of articles will serve as a valuable resource for the scientific community and contribute to the global effort to mitigate the impact of geminiviruses on agriculture and food security.

